# Effect of *Lactobacillus reuteri* on Gingival Inflammation and Composition of the Oral Microbiota in Patients Undergoing Treatment with Fixed Orthodontic Appliances: Study Protocol of a Randomized Control Trial

**DOI:** 10.3390/pathogens11020112

**Published:** 2022-01-18

**Authors:** Kevimy Agossa, Marie Dubar, Grégoire Lemaire, Alessandra Blaizot, Céline Catteau, Emmanuël Bocquet, Laurent Nawrocki, Emile Boyer, Vincent Meuric, Florence Siepmann

**Affiliations:** 1 Univ. Lille, Inserm, CHU Lille, U1008—Controlled Drug Delivery Systems and Biomaterials, F-59000 Lille, France; florence.siepmann@univ-lille.fr; 2 Department of Periodontology, School of Dentistry, University of Lille, Place de Verdun, F-59000 Lille, France; marie.dubar@univ-lille.fr (M.D.); dr.lemaire.59@gmail.com (G.L.); 3 Univ. Lille, Inserm, CHU Lille, UMR-S 1172, F-59000 Lille, France; 4 Department of Dental Public Health, School of Dentistry, CHU Lille, Univ. Lille, F-59000 Lille, France; alessandra.blaizot@univ-lille.fr (A.B.); celine.catteau@univ-lille.fr (C.C.); 5 Department of Orthodontics, School of Dentistry, CHU Lille, Univ. Lille, F-59037 Lille, France; emmanuelle.bocquet@univ-lille.fr; 6 Department of Oral Surgery, School of Dentistry, CHU Lille, Univ. Lille, F-59037 Lille, France; Laurent.NAWROCKI@chru-lille.fr; 7 INSERM, INRAE, Université de Rennes 1, CHU de Rennes, Nutrition Metabolisms and Cancer, F-35000 Rennes, France; emile.boyer@gmail.com (E.B.); vincent.meuric@univ-rennes1.fr (V.M.); 8 Teaching Hospital Pontchaillou, 2 rue Henri le Guilloux, F-35033 Rennes, France

**Keywords:** probiotics, adolescents, orthodontic treatment, prevention

## Abstract

The effect of probiotics in improving or maintaining oral health in orthodontic patients is understudied. The aim of this study is to evaluate the effect of probiotic administration in addition to tooth brushing on clinical gingival inflammation, plaque formation, subgingival microbiota composition, and salivary biomarkers of inflammation in adolescents with fixed orthodontic appliances. The present study is a 6-month, double-blind, two-arm, placebo-controlled, single-center trial, in which 116 adolescent volunteers aged 12–16 years will be recruited from the patients of the orthodontics clinic of the University Hospital of Lille, France. Subjects who meet the eligibility criteria will be allocated to one of the following groups: (i) control: two placebo lozenges per day for 90 days together with regular oral hygiene, (ii) test: two probiotic lozenges per day for 90 days together with regular oral hygiene. Clinical assessment and biological sample collection will be performed at baseline, 3 and 6 months. In addition, compliance outcomes and adverse events will be monitored.

## 1. Introduction

Fixed orthodontic appliances (FOA) including brackets, orthodontic bands, and wires are commonly used in orthodontic therapy to correct dental malposition. These fixed appliances, left in place for a prolonged duration, facilitate the accumulation of dental biofilm and make tooth brushing more challenging and time-consuming [1,2]. The persistence and accumulation of biofilm alter the composition of the supra- and subgingival microbiota. Thus, orthodontic patients present significant qualitative and quantitative differences in supra- and subgingival plaque compared to subjects without orthodontic appliances [3]. A recent study indicated a significant increase in *Bacteroidetes* and *Saccharibacteria* (formally TM7) and decrease in Proteobacteria and Actinobacteria over time, in both plaque and saliva [4]. At genus level, an increase in relative abundance of obligate anaerobes—including periodontal pathogens—was observed, which is likely to contribute to the increased prevalence of gingivitis during orthodontic treatment [5]. Importantly, these alterations in the composition of the oral microbiota would only be partially reversed even two years after the removal of fixed orthodontic appliances [6,7]. In this respect, supporting interventions for daily oral hygiene and oral health maintenance are needed for the favorable outcome of orthodontic treatment.

Probiotics are live organisms that confer health benefits to the host when administered in adequate amounts [8]. Since it is recognized that changes in the composition of dental biofilm play a key role in the initiation and progression of periodontal diseases, the administration of probiotic strains has been proposed to be useful in the prevention and treatment of these diseases [9,10,11,12]. *Lactobacillus* and *Bifidobacterium* are the most frequently described probiotics among which *Lactobacillus reuteri* (*L. reuteri*) is one of the most studied strains [13]. In vitro, probiotic strains can modulate the immuno-inflammatory response and are effective against oral pathogens through direct (adhesion or nutritive competitions and/or the secretion of antimicrobial molecules) and indirect (environmental modifications) mechanisms [13]. While a growing number of clinical studies have reported the use of probiotic to prevent or treat gingivitis and periodontitis, there is still a lack of clear evidence regarding their benefits [14,15,16,17,18,19].

From an ecological point of view, the origin of periodontal disease is a disruption of resilience caused by a “shift regime” (i.e., large, abrupt, persistent changes in function and structure) [20] within the normally stable resident oral microbiome [21]. The placement of fixed orthodontic appliances may lead to such changes, thus facilitating the establishment of local disease by promoting the accumulation of food debris and bacterial proliferation [1,2]. The composition of the microbiota associated with periodontal disease is complex and may include species from different phyla such as *Bacteroidetes*, *Firmicutes*, *Proteobacteria*, *Spirochaetes* and *Synergistetes and Saccharibacteria* [22]. The pathogenesis of periodontitis is also characterized by the high complexity of the immune-inflammatory mechanisms involved. A large number of signaling molecules are involved in the regulation of the host response and some are used as biomarkers of disease activity, progression, or resolution. This includes chemokines (e.g., IL-8, MIP-1, MCP-1, RANTES), cytokines (e.g., IL-1, TNF-α, INF-γ), arachidonic acid derivatives (e.g., PGE2), and proteases (e.g., matrix metalloproteinases, cathepsins) [23,24,25,26]. In this context, the use of orally active probiotics is expected to help in improving the resilience of the oral microbiome [27,28]. Probiotics produce compounds, including bacteriocins, that could contribute to the reduction of bacterial biofilms. Probiotic bacteria also prevent colonization by new pathogens by a firm adhesion to the surfaces of the oral cavity. Through this process, bacterial aggregation and co-aggregation occur, resulting in a new microbial balance and a healthy biofilm [29]. Furthermore, probiotics have been shown to modulate natural and acquired immune responses, via downregulation of inflammatory cascades by various mechanisms [30]. In vitro, the findings tend towards a modulation in the expression and/or production of IL-1β, TNFα, PGE2, TLR2-4, IL-8 and IL-10 by *Lactobacilli* and *Bifidobacteria* used in mono- or co-infection with periodontal pathogens on human gingival epithelial cells or fibroblasts [14]. Kobayashi et al. reported that oral administration of *Lactobacillus gasseri* was effective in preventing *Porphyromonas gingivalis*-induced periodontal disease in mice [31]. Data from clinical studies have shown a significant decrease in the prevalence of Gram-negative periodontal pathogens, including *Porphyromonas gingivalis*, *Aggregatibacter actinomycetemcomitans* and *Tannerella forsythia*, as well as a significant decrease in the level of proinflammatory cytokines in gingival crevicular fluid, after repeated consumption of probiotic *Lactobacillus* species [32,33,34]. Shimauchi et al. reported a decrease in the amount of biofilm on dental surfaces (reduction in plaque index) and an improvement in gingival inflammation in smokers after a probiotic intervention using *Lactobacillus salivarius* [35]. Other studies have shown an improvement in gingival inflammation with probiotics, but no effect on the amount of plaque. This could be explained by a direct inhibitory effect of probiotics on competing inflammophilic periodontopathogens and/or a probiotic-induced downregulation of an excessive proinflammatory host response towards them [36,37].

Two recent systematic reviews addressing the effect of probiotics in improving or maintaining gingival health in orthodontic patients with fixed appliances have only identified 4 studies which were all focused on the short-term (1 month maximum) effect of probiotics [38,39]. In two studies, probiotics were not effective in reducing plaque (PI) and gingivitis scores (GI) [40,41], while in the other two PI and GI were improved in patients receiving probiotics [42,43]. Other studies have investigated the influence of probiotics on the composition of the oral microbiota, but the effect on the number of cariogenic or periodontal pathogens remains inconclusive [38,39]. Therefore, the therapeutic potential of oral probiotics in orthodontic patients is yet to be investigated in high-quality studies of longer durations of intervention and follow-up.

The aim of this study is to evaluate the effect of twice-daily administration of probiotics in addition to tooth brushing on gingival inflammation in adolescents with fixed orthodontic appliances.

## 2. Materials and Method

### 2.1. Research Hypothesis and Objectives

The gingival index (Loë Silness) [44] would be lower in adolescents with fixed orthodontic appliances receiving probiotics in addition to oral hygiene compared to those practicing oral hygiene alone.

#### 2.1.1. Primary Objective

Evaluation of the gingival index at 3 months: to compare the effect of twice-daily administration of probiotic lozenges during 3 months in addition to tooth brushing versus tooth brushing alone on gingival index at 3 months in adolescents undergoing treatment with fixed orthodontic appliances.

#### 2.1.2. Secondary Objectives

To compare the effect of adjunctive probiotics on the following parameters:

- Gingival index at 6 months (i.e., 3 months after cessation of the lozenges intake).
- Extent and severity of gingivitis (at 3 and 6 months).
- Plaque accumulation on dental surfaces (at 3 and 6 months).
- Relative abundance of bacterial taxa at the phylum level in the supra and sub gingival microbiota (at 3 and 6 months).
- Level of salivary biomarkers of inflammation (at 3 and 6 months).

### 2.2. Study Design

The present study is a single-center, randomized, double blinded, two-armed, placebo-controlled trial conducted at Lille University-Hospital (CHU Lille, France). The study protocol was developed according to the Standard Protocol Items Recommendations for Interventional Studies (SPIRIT) and registered in the ClinicalTrials.gov (accessed on 16 December 2021) (NCT04634201) database.

### 2.3. Ethical Considerations

This trial will be conducted in accordance with the principles of the Declaration of Helsinki for studies involving humans. The protocol has been approved by the Institutional Review Board of OUEST IV (France)—CPP 1360 HPS2 (N° RIPH: 20.11.20.40523). All eligible volunteers will be informed (orally and written) about the nature, potential risks, and benefits of their participation in this study, and they will sign an informed consent.

### 2.4. Sample Size Calculation

The minimal sample size to assure adequate power for this study was calculated based on the variation of the mean gingival index at 6 months after treatment [45]. Considering a difference of 0.3 in the gingival index value at 6 months between the test and control groups, a significant level of 5% (two-sided test) and an attrition rate of 10%, 58 subjects should be included in each group to provide a power of 80%, i.e., a total of 116 patients.

### 2.5. Subject Population and Inclusion/Exclusion Criteria

Volunteers will be recruited from the outpatients of the orthodontics clinic of the University Hospital of Lille, France. Subjects will be selected according to the following criteria: (i) adolescents aged between 12–16 years, (ii) in good systemic health and proficient in tooth brushing, (iii) eligible for full arch upper and lower fixed labial orthodontic appliance treatment, (iv) with no active untreated periodontitis or evolutive gingival recession; (v) willing and able to comply with the study protocol and whose parents/guardians have given their consent. Exclusion criteria include (vi) inability to obtain informed consent, the presence of (vii) allergies, sensitivities or food intolerance to any of the components of the probiotic lozenges or placebo, (viii) systemic diseases that could affect the periodontal status (e.g., diabetes, immunological disorders), (ix) chronic use of probiotics or food supplements, and (x) use of antibiotic therapy in the previous month/ need for antibiotic premedication for dental treatment and long-term intake of anti-inflammatory drugs.

### 2.6. Interventions

**Figure 1:**
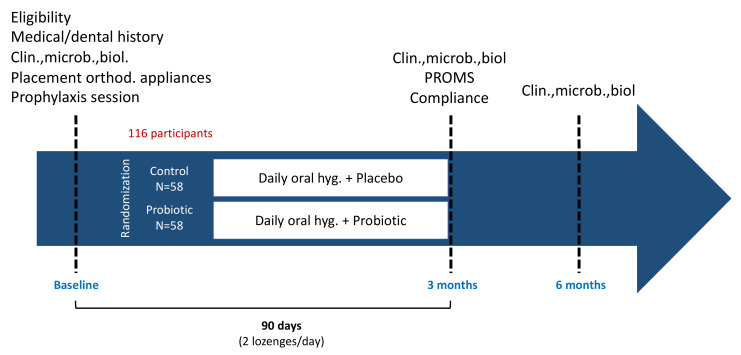
Experimental design of the study. Clin, clinical assessment; microb, microbiological assessment; biol, biological assessment; PROMS, patient-reported outcomes measures; hyg, hygiene; orthod, orthodontic.

At baseline, demographic, oral and general health information will be collected. Subsequently, a clinical periodontal examination, as well as supra and sub gingival biofilm and saliva sampling will be performed. A professional mechanical plaque removal will be provided using air-polishing combined with manual and ultrasonic scalers and patients will receive oral hygiene instruction (OHI), including tooth brushing and inter-proximal dental hygiene using interdental brushes. Subsequently, patients will be randomly allocated to one of the following groups: (i) control: two placebo lozenges a day for 90 days together with regular oral hygiene, (ii) test: two probiotic lozenges a day for 90 days together with regular oral hygiene (Figure 1 and Table 1). The administration of probiotics and placebo will start on the day of placement of fixed orthodontic appliances. The probiotic (or placebo) will be delivered for 4 weeks and will be refilled periodically. The probiotic studied (Gum^®^ PerioBalance^®^) is commercially available in France. Probiotic and placebo are prepared by Bio-Gaia AB (Stockholm, Sweden). Each lozenge of probiotic contains two strains of probiotic lactic acid bacteria of human origin: *L. reuteri* DSM 17938 and *L. reuteri* ATCC (PTA 5289), each at a minimum amount of 1 × 10^8^ CFU per lozenge. The probiotic will be used according to the manufacturer’s recommendations at a dosage of 2 lozenges/day. The composition and packaging of the placebo will be identical in all aspects to the probiotic but do not contain *L. reuteri*. The lozenges should be stored at a temperature below 25 °C. Patients will be asked to avoid the concomitant use of antibiotics or antiseptic mouthwashes. However, if the use of these medications is required by prescription, the principal investigator should be informed.

### 2.7. Primary and Secondary Outcomes Variables

The primary outcome is the difference for gingival index at 6 months post-treatment [46,47]. Secondary outcomes include:

Difference between baseline and 3–6 months post-therapy for bleeding on probing (BOP) [48];

Mean number and percentage of participants with different BOP thresholds (e.g., BOP < 30%, BOP ≥ 30%), and gingival index thresholds (e.g., GI ≤ 1, GI ≥ 2) 3 and 6 months post therapy;

Difference between baseline and 3–6 months post-therapy for plaque score [49];

Differences between groups in occurrence of adverse events, compliance [50], and patient-reported outcomes [50];

Difference in the relative abundance of bacterial taxa in subgingival microbiota (alpha and beta diversity, Bray–Curtis distance and UniFrac).

Levels of 20 cytokines and inflammatory mediators in saliva at 3 and 6 months after therapy.

### 2.8. Monitoring of Compliance Patient-Related Outcomes and Adverse Events (3 and/or 6 Months)

The daily intake of the lozenges will be encouraged by the orthodontist at the patient’s regular appointments approximately once every 4–6 weeks. Patients will be asked to return empty packages and unused lozenges to the investigator at the end of the 3-month treatment period to enable compliance to be monitored. In addition, patients will complete a weekly record of missed treatments in a diary. At 3 months, participants will complete a questionnaire to report their subjective evaluation of lozenges (taste/pleasantness, feeling of “clean teeth”) and the occurrence of any adverse effects [51]. In addition, the presence of any potential side effects in the oral cavity (e.g., lesions of the oral mucosa) will be assessed at the 3 and 6 month visits.

### 2.9. Clinical Examination (Baseline, 3 and 6 Months)

Two calibrated examiners (KA, MD) will perform the clinical assessment blinded to the patient’s allocation at baseline, 3, and 6 months as well as the biofilm, saliva and GCF samples at baseline and 3 months. As much as possible, follow-up visits (3 and 6 months) will be planned together with periodic orthodontic treatment check-ups to minimize the burden of the study on the participants and to reduce the risk of loss to follow-up. The following periodontal parameters will be evaluated: Löe and Silness gingival index [46], bleeding on probing score (BOP) using a North Carolina periodontal probe (Hu-Friedy, Chicago, IL, USA) [48], Visible plaque (O’leary index) [49].

### 2.10. Microbiological Monitoring (Baseline, 3 and 6 Months)

Samples of supra gingival biofilm will be collected on the vestibular, occlusal, and lingual surfaces of the tooth with a sterile swab. Subgingival biofilm will be collected (4 sites per patient) on selected sites located on non-contiguous teeth (preferably 1 site per quadrants) at baseline, 3 and 6 months. after removal of the supragingival plaque. The subgingival samples will be collected using sterile paper points (Henry Schein, France) inserted into the most apical part of the sulcus for 30 s. All the samples (supra gingival dental plaque collected on tooth surface using a sterile swab, and sub gingival plaque collected using sterile paper points inserted in the sulcus) will be immediately placed in separate Eppendorf tubes and kept at −80 °C until analysis. Oral microbiota composition will be evaluated as described in a previous paper [52]. Microbiological analysis will be performed at Inserm U1241 NuMeCan (Nutrition Metabolisms and Cancer), University of Rennes. Briefly, taxonomy at the genus level will be assigned using the “Visualization and Analysis of Microbial Population Structures” (VAMPS) analysis pipeline web tool. Default parameters will be used for assignment at the genus level with the “Ribosomal Database Project” classification. Alpha rarefaction and diversity will be evaluated using the Observed Species metric (Sobs) and the Shannon–Weaver index. Beta diversity will be calculated using Bray–Curtis and UniFrac distances.

### 2.11. Salivary Biomarkers Monitoring (Baseline, 3 and 6 Months)

Saliva samples will be taken from each patient using a cotton swab (Salivette^®^, Sarstedt, Marnay, France) according to the manufacturer’s instructions. The patients will be instructed to use the device to collect their saliva on the morning of the day of the visit before eating, drinking, or any oral hygiene procedure. Briefly, the swab should be kept in the mouth without touching it for 2–3 min until it is soaked and then replaced in its tube and returned to the department during the day with temporary storage in the refrigerator. Once received, the tube will be immediately centrifuged at 1000× *g* for 1 min and frozen at −80 °C until analysis. An immunology multiplex assay will be used to detect the level of selected cytokines and inflammatory mediators including IL-1β, TNF-α, IL-6, IL-8, IL-10 from the saliva samples using the ProcartaPlex Human Inflammation Panel (20 Plex) (eBioscience) according to manufacturer’s instructions [53].

### 2.12. Randomization et Data Management

Randomization will be performed using a randomization table generated by the software SAS (SAS Institute, Cary, NC, USA) in a balanced parallel design (1:1) using variable block sizes. This table will be implemented directly in an electronic case report form. A file describing the random procedure is confidentially classified at the Clinical Research Department (DRICI) in the University Hospital of Lille.

The individual data collected during the study will be reported on a source document and then recorded in a computer database via Clinsight^®^ software certified for data management in clinical trials according to the recommendations of the FDA (Food and Drug Administration). All data will be stored on a secured server. Before locking the database, the data will be monitored according to the coherence criteria agreed upon with the investigator. The data will be processed in accordance with the MR 001 reference methodology described by the CNIL (French data protection authority). Access to the data will be restricted to persons directly involved in the study. Data may only be modified by an investigator participating in the study or a staff member participating in the study. The data will be stored for a period of at least 15 years from the end of the research.

### 2.13. Statistical Analysis

All statistical analyses will be performed in an independent and blind way to the allocated group, using SAS software (SAS Institute, Cary, NC, USA). The level of significance will be set at 5%. The data will be evaluated using intention-to-treat analysis with the last observation carried forward. To ensure comparability between groups, the characteristics of the patients at inclusion will be described for each of the two study groups. Quantitative variables will be described by the mean and standard deviation in case of normality of the distribution, or by the median and interquartile range otherwise. The normality of the distributions will be confirmed by the Shapiro–Wilk test. The qualitative variables will be described by the numbers and percentages of each variable.

The variation of the gingival index (difference between baseline and 6-month measurement) will be estimated and compared between the two groups using a constrained longitudinal data analysis (cLDA) model as proposed by Liang and Zeger [54]. In this mixed linear model, the initial and post-treatment values will be coded as dependent variables, with a restriction on a value common to both groups at inclusion in order to control for the change between groups with respect to the baseline value and to take into account all available measures [55]. The mean change at 6 months between the groups (adjusted on baseline values) will be estimated by the time x group interaction term. In case of deviation from normality, the difference between the baseline and 6 month measures will be calculated and compared between groups using a non-parametric analysis of covariance adjusted on the baseline measure [56,57]. The variation between baseline and 3–6 months for BOP, plaque score, alpha/beta diversity index and levels of inflammatory mediators will be analyzed by the cLDA method described above for the primary outcome. A stepwise logistic regression analysis will be performed to investigate the impact of predictor variables on the clinical endpoint for treatment (e.g., BOP < 10%, GI ≤ 1). Descriptive analyses will be performed for the number of adverse effects, compliance and patient related outcomes.

The similarity of microbiota according to different clinical conditions will be analyzed using the Bray–Curtis and UniFrac distances. Taxonomic profiles will be analyzed using weighted linear discriminant analysis (LEfSe) and taxa will be used to generate correlation matrices (calculated using 4 similarity measures, namely Spearman and Pearson correlations and non-parametric Bray–Curtis and Kull–Black–Leibler distances) with the observed clinical outcomes [58].

Missing data on the primary outcome (irrespective of the reason for missing data) will be imputed under the “missing at random” (MAR) assumption using the chained equations method with 10 imputations. The variables used for the imputation model will be patient characteristics at randomization and treatment group. Quantitative variables will be imputed by the predictive mean matching method and qualitative variables by logistic regression models (binary, ordinal, or multinomial depending on the number and order of the modalities). Rubin’s rules will be used to combine the estimates obtained in each table of imputed data [59].

## 3. Discussion

As the health and expectations of the population are improving, orthodontic treatment needs are increasing. The prevalence of malocclusions in children and adolescents varies between populations but remains high overall, estimated to range from 39 to 93% [60]. According to a previous survey in the UK, approximately one-third of children would benefit from orthodontic treatment [61,62]. Similar results have been reported in other European countries [63,64,65]. Maintaining a high standard of oral hygiene during orthodontic treatment is one of the most challenging aspects of clinical orthodontics [66]. Indeed, patients undergoing orthodontic treatments with fixed appliances typically have a higher accumulation of biofilm which exposes them to a higher risk of caries and gingivitis that can impair the course and outcome of treatment [5].

The use of probiotic microorganisms in the management of periodontal disease is a promising approach and a rapidly evolving field. A recent comprehensive review identified 36 RCTs published between 2009 and 2021 addressing this issue, with slightly under 50% focusing on the management of gingivitis [67]. Overall, the administration of probiotics, either as an adjunct to mechanical plaque control or as a single intervention, has yielded heterogeneous results. Some studies have shown a benefit of probiotic consumption in reducing the level of gingival inflammation and the amount of plaque [68,69], while others have failed to demonstrate any additional clinical benefit [70,71,72]. However, it is worth mentioning that the potential effect of probiotics seems to be more substantial among patients who cannot undergo strict mechanical plaque control or who concomitantly present an altered systemic inflammatory response. In patients with strict plaque control, the additional benefit of probiotics, if any, would be very limited and difficult to detect clinically [36,37,73]. From this point of view, the study population is clearly relevant. Adolescents with fixed orthodontic appliance present a higher risk of gingival inflammation due to the combined effect of the lack of access to oral hygiene related to orthodontic appliances, but also due to the elevation of sex steroid hormones that can generate an increased inflammatory response to plaque accumulation [74].

Some methodological choices were made for this study that should allow the provision of robust data and overcome the limitations of the few available studies on the benefit of probiotics during orthodontic treatment [38]. First, the administration of probiotics over a period of 3 months will allow sufficient time for colonization of the oral cavity by the probiotics and thus increase the chances of detecting a potential effect. Indeed, it has been observed that the effect of probiotics on the composition of the oral microbiota would not be detectable before 6 weeks of administration [33]. This could explain why previous studies have failed to demonstrate short-term efficacy of probiotics compared to placebo [42,75]. The planned microbiological analyses will verify whether the clinical outcomes are correlated with a bacterial shift. Secondly, the total duration of the study is 6 months, i.e., 3 months after the completion of the probiotics administration to allow us to evaluate the existence of any remaining effect of the probiotics after their discontinuation. Given that orthodontic treatments can last 20 months on average [76], patient compliance with daily intake of probiotic lozenges for the entire duration of the treatment is unrealistic. Third, it should be noted that at the beginning of the study, participants will benefit from a professional prophylaxis session since all orthodontic patients are expected to practice daily home oral hygiene as a standard of care. However, no further professional tooth cleaning or oral hygiene instruction will be provided during the study period to interfere as little as possible with the usual mechanical plaque control of the participants. Finally, the focus on a specific age group and a single type of probiotic could be considered a limitation to the extent to which the study results can be extrapolated. However, it should be kept in mind that children and young patients represent the vast majority of patients treated in orthodontics. Furthermore, *Lactobacillus reuteri* strains (DSM17938; ATCC PTA 5289) are well characterized in vitro and are the most frequently evaluated probiotic bacteria in oral clinical trials [13,67].

In conclusion, this study is expected to provide some reliable evidence regarding the effects of probiotics on gingival inflammation in patients undergoing treatment with fixed orthodontic appliances. This study should also provide new insight to better prevent periodontal disease and optimize orthodontic therapy.

## 4. Trial Status

This is the 1.1 version of the protocol dated 15 January 2021. Recruitment should start on January 2022, and we anticipate recruitment will end on January 2023.

## Figures and Tables

**Table 1:** Schedule of volunteers’ enrolment, interventions, and assessments according to the SPIRIT statement.

	Enrolment	Allocation	Post-Allocation		
TIMEPOINT	T-1		Intervention (T0)	3 Month Follow-Up (T1)	6 Month Follow-Up (T2)
ENROLMENT:					
Elligibility screen	X				
Informed consent	X				
Medical/dental history	X				
Allocation		X			
INTERVENTIONS:					
Orthodontic appliances			X		
Prophylaxis session			X		
Probiotic/placebo delivery			X		
ASSESSMENTS:					
Clinical		X		X	X
Microbiological		X		X	X
Biological		X		X	X
PROMS ^1^				X	
Compliance				X	

^1^ Patient-reported outcomes measures.

## Data Availability

No new data were created or analyzed in this study. Data sharing is not applicable to this article.

## References

[1] Ren Y., Jongsma M.A., Mei L., Van Der Mei H.C., Busscher H.J. (2014). Orthodontic treatment with fixed appliances and biofilm formation—A potential public health threat?. Clin. Oral Investig..

[2] Jiang Q., Li J., Mei L., Du J., Levrini L., Abbate G.M., Li H. (2018). Periodontal health during orthodontic treatment with clear aligners and fixed appliances. J. Am. Dent. Assoc..

[3] Contaldo M., Lucchese A., Lajolo C., Rupe C., Di Stasio D., Romano A., Petruzzi M., Serpico R. (2021). The Oral Microbiota Changes in Orthodontic Patients and Effects on Oral Health: An Overview. J. Clin. Med..

[4] Kado I., Hisatsune J., Tsuruda K., Tanimoto K., Sugai M. (2020). The impact of fixed orthodontic appliances on oral microbiome dynamics in Japanese patients. Sci. Rep..

[5] Müller L.K., Jungbauer G., Jungbauer R., Wolf M., Deschner J. (2021). Biofilm and orthodontic therapy. Monogr. Oral Sci..

[6] Ghijselings E., Coucke W., Verdonck A., Teughels W., Quirynen M., Pauwels M., Carels C., van Gastel J. (2013). Long-term changes in microbiology and clinical periodontal variables after completion of fixed orthodontic appliances. Orthod. Craniofacial Res..

[7] Kim K., Jung W.-S., Cho S., Ahn S.-J. (2015). Changes in salivary periodontal pathogens after orthodontic treatment: An in vivo prospective study. Angle Orthod..

[8] Dhansekaran D., Sankaranarayanan A., FAO, WHO (2006). Probiotics in food: Health and nutritional properties and guidelines for evaluation. Advances in Probiotics: Microorganisms in Food and Health.

[9] Meurman J.H., Stamatova I. (2007). Probiotics: Contributions to oral health. Oral Dis..

[10] Meurman J.H. (2005). Probiotics: Do they have a role in oral medicine and dentistry?. Eur. J. Oral Sci..

[11] Saïz P., Taveira N., Alves R. (2021). Probiotics in Oral Health and Disease: A Systematic Review. Appl. Sci..

[12] Mahasneh S.A., Mahasneh A.M. (2017). Probiotics: A Promising Role in Dental Health. Dent. J..

[13] Routier A., Blaizot A., Agossa K., Dubar M. (2021). What do we know about the mechanisms of action of probiotics on factors involved in the pathogenesis of periodontitis? A scoping review of in vitro studies. Arch. Oral Biol..

[14] Martin-Cabezas R., Davideau J.L., Tenenbaum H., Huck O. (2016). Clinical efficacy of probiotics as an adjunctive therapy to non-surgical periodontal treatment of chronic periodontitis: A systematic review and meta-analysis. J. Clin. Periodontol..

[15] Ikram S., Hassan N., Raffat M.A., Mirza S., Akram Z. (2018). Systematic review and meta-analysis of double-blind, placebo-controlled, randomized clinical trials using probiotics in chronic periodontitis. J. Investig. Clin. Dent..

[16] Hu D., Zhong T., Dai Q. (2021). Clinical efficacy of probiotics as an adjunctive therapy to scaling and root planning in the management of periodontitis: A systematic review and meta-analysis of randomized controlled trails. J. Evid. Based Dent. Pract..

[17] Akram Z., Shafqat S.S., Aati S., Kujan O., Fawzy A. (2020). Clinical efficacy of probiotics in the treatment of gingivitis: A systematic review and meta-analysis. Aust. Dent. J..

[18] Liu J., Liu Z., Huang J., Tao R. Effect of probiotics on gingival inflammation and oral microbiota: A meta-analysis. Oral Dis..

[19] Barboza E.P., Arriaga P.C., Luz D.P., Montez C., Vianna K.C. (2020). Systematic review of the effect of probiotics on experimental gingivitis in humans. Braz. Oral Res..

[20] Folke C., Carpenter S., Walker B., Scheffer M., Elmqvist T., Gunderson L., Holling C.S. (2004). Regime shifts, resilience, and biodiversity in ecosystem management. Annu. Rev. Ecol. Evol. Syst..

[21] Rosier B.T., Marsh P.D., Mira A. (2018). Resilience of the Oral Microbiota in Health: Mechanisms That Prevent Dysbiosis. J. Dent. Res..

[22] Pérez-Chaparro P.J., Gonçalves C., Figueiredo L.C., Faveri M., Lobão E., Tamashiro N., Duarte P., Feres M. (2014). Newly identified pathogens associated with periodontitis: A systematic review. J. Dent. Res..

[23] Elkaim R., Werner S., Kocgozlu L., Tenenbaum H. (2008). *P. gingivalis* Regulates the Expression of Cathepsin B and Cystatin C. J. Dent. Res..

[24] Di Benedetto A., Gigante I., Colucci S., Grano M. (2013). Periodontal disease: Linking the primary inflammation to bone loss. Clin. Dev. Immunol..

[25] Buduneli N., Kinane D.F. (2011). Host-derived diagnostic markers related to soft tissue destruction and bone degradation in periodontitis. J. Clin. Periodontol..

[26] Agossa K., Morand D.N., Tenenbaum H., Davideau J.L., Huck O. (2015). Systemic applications of anti-inflammatory agents in periodontal treatment. Clin. Anti-Inflamm. Anti-Allergy Drugs.

[27] Anusha R.L., Umar D., Basheer B., Baroudi K. (2015). The magic of magic bugs in oral cavity: Probiotics. J. Adv. Pharm. Technol. Res..

[28] Rastogi P., Saini H., Dixit J., Singhal R. (2011). Probiotics and oral health. Natl. J. Maxillofac. Surg..

[29] Mishra S., Rath S., Mohanty N. (2020). Probiotics—A complete oral healthcare package. J. Integr. Med..

[30] Gill H., Prasad J. (2008). Probiotics, immunomodulation, and health benefits. Adv. Exp. Med. Biol..

[31] Kobayashi R., Kobayashi T., Sakai F., Hosoya T., Yamamoto M., Kurita-Ochiai T. (2017). Oral administration of Lactobacillus gasseri SBT2055 is effective in preventing Porphyromonas gingivalis-accelerated periodontal disease. Sci. Rep..

[32] Teughels W., Durukan A., Ozcelik O., Pauwels M., Quirynen M., Haytac M.C. (2013). Clinical and microbiological effects of Lactobacillus reuteri probiotics in the treatment of chronic periodontitis: A randomized placebo-controlled study. J. Clin. Periodontol..

[33] Montero E., Iniesta M., Rodrigo M., Marín M.J., Figuero E., Herrera D., Sanz M. (2017). Clinical and microbiological effects of the adjunctive use of probiotics in the treatment of gingivitis: A randomized controlled clinical trial. J. Clin. Periodontol..

[34] Twetman S., Derawi B., Keller M., Ekstrand K., Yucel-Lindberg T., Stecksen-Blicks C. (2009). Short-term effect of chewing gums containing probiotic Lactobacillus reuteri on the levels of inflammatory mediators in gingival crevicular fluid. Acta Odontol. Scand..

[35] Shimauchi H., Mayanagi G., Nakaya S., Minamibuchi M., Ito Y., Yamaki K., Hirata H. (2008). Improvement of periodontal condition by probiotics with Lactobacillus salivarius WB21: A randomized, double-blind, placebo-controlled study. J. Clin. Periodontol..

[36] Schlagenhauf U., Rehder J., Gelbrich G., Jockel-Schneider Y. (2020). Consumption of Lactobacillus reuteri-containing lozenges improves periodontal health in navy sailors at sea: A randomized controlled trial. J. Periodontol..

[37] Schlagenhauf U., Jakob L., Eigenthaler M., Segerer S., Jockel-Schneider Y., Rehn M. (2016). Regular consumption of Lactobacillus reuteri-containing lozenges reduces pregnancy gingivitis: An RCT. J. Clin. Periodontol..

[38] Hadj-Hamou R., Senok A.C., Athanasiou A.E., Kaklamanos E.G. (2020). Do probiotics promote oral health during orthodontic treatment with fixed appliances? A systematic review. BMC Oral Heal..

[39] Pietri F.K., Rossouw P.E., Javed F., Michelogiannakis D. (2020). Role of Probiotics in Oral Health Maintenance Among Patients Undergoing Fixed Orthodontic Therapy: A Systematic Review of Randomized Controlled Clinical Trials. Probiotics Antimicrob. Proteins.

[40] Benic G.Z., Farella M., Morgan X.C., Viswam J., Heng N.C., Cannon R.D., Mei L. (2019). Oral probiotics reduce halitosis in patients wearing orthodontic braces: A randomized, triple-blind, placebo-controlled trial. J. Breath Res..

[41] Habib S. (2016). Assessment of the Therapeutic Potential of a Dental Probiotic in Orthodontic Patients Affected by Gingivitis: A Randomized Controlled Trial. Master’s Thesis.

[42] Kohar M., Emmanuel V., Astuti L. (2015). Comparison between probiotic lozenges and drinks towards periodontal status improvement of orthodontic patients. Dent. J. (Maj. Kedokt. Gigi).

[43] Shah S.S., Nambiar S., Kamath D., Suman E., Unnikrishnan B., Desai A., Mahajan S., Dhawan K.K. (2019). Comparative evaluation of plaque inhibitory and antimicrobial efficacy of probiotic and chlorhexidine oral rinses in orthodontic patients: A randomized clinical trial. Int. J. Dent..

[44] Löe H. (1967). The Gingival Index, the Plaque Index and the Retention Index Systems. J. Periodontol..

[45] Alanzi A., Honkala S., Honkala E., Varghese A., Tolvanen M., Söderling E. (2018). Effect of Lactobacillus rhamnosus and Bifidobacterium lactis on gingival health, dental plaque, and periodontopathogens in adolescents: A randomised placebo-controlled clinical trial. Benef. Microbes.

[46] Löe H., Silness J. (1963). Periodontal disease in pregnancy I. Prevalence and severity. Acta Odontol. Scand..

[47] Benamghar L., Leclercq M.H., Bourgeois D., McCombie B.J., Barmes D.E. (1994). Standard descriptive tables in WHO Oral Health epidemiological studies. World Health Stat. Q..

[48] Ainamo J., Bay I. (1975). Problems and proposals for recording gingivitis and plaque. Int. Dent. J..

[49] O’Leary T.J., Drake R.B., Naylor J.E. (1972). The plaque control record. J. Periodontol..

[50] Hedayati-Hajikand T., Lundberg U., Eldh C., Twetman S. (2015). Effect of probiotic chewing tablets on early childhood caries-a randomized controlled trial. BMC Oral Health..

[51] Herrera D., Escudero N., Perez L., Otheo M., Canete-Sanchez E., Perez T., Alonso B., Serrano J., Palma J.C., Sanz M. (2018). Clinical and microbiological effects of the use of a cetylpyridinium chloride dentifrice and mouth rinse in orthodontic patients: A 3-month randomized clinical trial. Eur. J. Orthod..

[52] Boyer E., Le Gall-David S., Martin B., Fong S.B., Loréal O., Deugnier Y., Bonnaure-Mallet M., Meuric V. (2018). Increased transferrin saturation is associated with subgingival microbiota dysbiosis and severe periodontitis in genetic haemochromatosis. Sci. Rep..

[53] Lundmark A., Hu Y.O.O., Huss M., Johannsen G., Andersson A.F., Yucel-Lindberg T. (2019). Identification of Salivary Microbiota and Its Association With Host Inflammatory Mediators in Periodontitis. Front. Cell. Infect. Microbiol..

[54] Liang K.Y., Zeger S. (2000). Longitudinal data analysis of continuous and discrete responses for pre-post designs. Sankhya Ind. J. Stat. Ser. B..

[55] Liu G.F., Lu K., Mogg R., Mallick M., Mehrotra D.V. (2009). Should baseline be a covariate or dependent variable in analyses of change form baseline in clinical trials?. Stat. Med..

[56] Vickers A.J. (2005). Parametric versus non-parametric statistics in the analysis of randomized trials with non-normally distributed data. BMC Med. Res. Methodol..

[57] Conover W.J., Iman R.L. (1982). Analysis of covariance using the rank transformation. Biometrics.

[58] Meuric V., Lainé F., Boyer E., Le Gall-David S., Oger E., Bourgeois D., Bouchard P., Bardou-Jacquet E., Turmel V., Bonnaure-Mallet M. (2017). Periodontal status and serum biomarker levels in HFE haemochromatosis patients. A case-series study. J. Clin. Periodontol..

[59] Rubin D.B. (1987). Multiple Imputation for Nonresponse in Surveys.

[60] Cenzato N., Nobili A., Maspero C. (2021). Prevalence of Dental Malocclusions in Different Geographical Areas: Scoping Review. Dent. J..

[61] Chestnutt I.G., Burden D.J., Steele J.G., Pitts N.B., Nuttall N.M., Morris A.J. (2006). The orthodontic condition of children in the United Kingdom, 2003. Br. Dent. J..

[62] Jawad Z., Bates C., Hodge T. (2015). Who needs orthodontic treatment? Who gets it? And who wants it?. Br. Dent. J..

[63] Bilgic F., Gelgor I.E., Celebi A.A. (2015). Malocclusion prevalence and orthodontic treatment need in central Anatolian adolescents compared to European and other nations’ adolescents. Dent. Press J. Orthod..

[64] Josefsson E., Bjerklin K., Lindsten R. (2007). Malocclusion frequency in Swedish and immigrant adolescents: Influence of origin on orthodontic treatment need. Eur. J. Orthod..

[65] Perillo L., Masucci C., Ferro F., Apicella D., Baccetti T. (2010). Prevalence of orthodontic treatment need in southern Italian schoolchildren. Eur. J. Orthod..

[66] Travess H., Roberts-Harry D., Sandy J. (2004). Orthodontics. Part 6: Risks in orthodontic treatment. Br. Dent. J..

[67] Schlagenhauf U., Jockel-Schneider Y. (2021). Probiotics in the management of gingivitis and periodontitis. Front. Dent. Med..

[68] Kuru B.E., Laleman I., Yalnizoglu T., Kuru L., Teughels W. (2017). The influence of a *bifidobacterium animalis* probiotic on gingival health: A randomized controlled clinical trial. J. Periodontol..

[69] Toiviainen A., Jalasvuori H., Lahti E., Gursoy U., Salminen S., Fontana M., Flannagan S., Eckert G., Kokaras A., Paster B. (2015). Impact of orally administered lozenges with Lactobacillus rhamnosus GG and Bifidobacterium animalis subsp. lactis BB-12 on the number of salivary mutans streptococci, amount of plaque, gingival inflammation and the oral microbiome in healthy adults. Clin. Oral Investig..

[70] Alkaya B., Laleman I., Keceli S., Ozcelik O., Haytac M.C., Teughels W. (2017). Clinical effects of probiotics containing Bacillus species on gingivitis: A pilot randomized controlled trial. J. Periodontal Res..

[71] Iniesta M., Herrera D., Montero E., Zurbriggen M., Matos A.R., Marin M.J., Sanchez-Beltran M.C., Llama-Palacio A., Sanz M. (2012). Probiotic effects of orally administered Lactobacillus reuteri-containing tablets on the subgingival and salivary microbiota in patients with gingivitis. A randomized clinical trial. J. Clin. Periodontol..

[72] Hallstrom H., Lindgren S., Yucel-Lindberg T., Dahlen G., Renvert S., Twetman S. (2013). Effect of probiotic lozenges on inflammatory reactions and oral biofilm during experimental gingivitis. Acta Odontol. Scand..

[73] Yuki O., Furutani C., Mizota Y., Wakita A., Mimura S., Kihara T., Ohara M., Okada Y., Okada M., Nikawa H. (2019). Effect of bovine milk fermented with Lactobacillus rhamnosus L8020 on periodontal disease in individuals with intellectual disability: A randomized clinical trial. J. Appl. Oral Sci..

[74] Trombelli L., Farina R. (2013). A review of factors influencing the incidence and severity of plaque-induced gingivitis. Minerva Stomatol..

[75] Benic G.Z. (2016). Biofilm Management with Oral probiotics in Patients with Fixed Orthodontic Appliances. Ph.D. Thesis.

[76] Tsichlaki A., Chin S.Y., Pandis N., Fleming P.S. (2016). How long does treatment with fixed orthodontic appliances last? A systematic review. Am. J. Orthod. Dentofac. Orthop..

